# Ageing exacerbates the adverse effects of respiratory muscle fatigue on vascular function, locomotor muscle fatigue and exercise performance in males

**DOI:** 10.1113/EP092897

**Published:** 2025-06-05

**Authors:** Valentin Mons, Colin Lavigne, Olivier Meste, Benjamin Mauroy, Gregory M. Blain

**Affiliations:** ^1^ Université Côte d'Azur, LAMHESS Nice France; ^2^ Université Côte d'Azur, CNRS, LJAD Nice France; ^3^ Université Côte d'Azur, Centre Vader Nice France; ^4^ Université Côte d'Azur Laboratoire, CNRS, I3S Nice France

**Keywords:** ageing, exercise, master athletes, mean arterial pressure, neuromuscular fatigue, performance, respiratory muscle fatigue, respiratory muscle metaboreflex

## Abstract

This study investigated the effect of respiratory muscle fatigue on cardiovascular function, locomotor muscle fatigue and exercise performance in young and master athletes, a model of successful ageing. Ten young male (YA, 27.4 ± 4.4 years) and 11 male master endurance athletes (MA, 65.0 ± 5.1 years) performed, on separate days, two constant workload cycling tests at 90% of peak power to exhaustion (CWT) following a fatiguing inspiratory loading task at 60% (ILT_60%_) and a sham task at 2% (ILT_2%_) of their maximal inspiratory pressure. On a third day, the sham task was replicated but CWT was interrupted at the time equal to that performed during CWT_ILT60%_ (CWT_ILT2%–ISO_). Quadriceps fatigue was assessed by changes in maximal voluntary isometric contraction (MVC), potentiated twitch force (QT_SINGLE_) and voluntary activation (VA) from 15 s to 15 min post‐exercise. Mean arterial pressure (MAP) was measured using finger pulse photoplethysmography. Blood flow (*Q̇*_L_) and limb vascular conductance (LVC) were measured using Doppler ultrasound. During ILT_60%_, MA demonstrated reduced *Q̇*_L_ (*P* = 0.036), a greater increase in MAP (*P *< 0.001) and a larger decrease in LVC (*P *= 0.044) compared to YA. During CWT_ILT60%_, MA experienced a larger decrease in time to exhaustion (−39.7 ± 14.0%) than YA (−15.5 ± 13.9%, *P* = 0.010). Exercise‐induced reductions in MVC and QT_SINGLE_ (both *P *< 0.039) were also more pronounced during CWT_ILT60%_ compared to CWT_ILT2%–ISO_ in MA. Ageing exacerbates the adverse effects of respiratory muscle fatigue on limb vascular function and locomotor muscle fatigue during subsequent exercise, resulting in greater impairments in exercise performance.

## Abstract (highlights)

- **What is the central question of this study?**
  What is the effect of respiratory muscle fatigue on cardiovascular function, locomotor muscle fatigue and exercise performance in young and master athletes?
- **What is the main finding and its importance?**
  Ageing exacerbates the respiratory metaboreflex – the pressor response triggered by respiratory muscle fatigue – resulting in greater increase in mean arterial pressure and reduction in limb vascular conductance. Similar pre‐existing respiratory muscle fatigue accelerates locomotor muscle fatigue and more significantly impairs exercise performance in master athletes compared to younger counterparts. This underscores the respiratory system's growing contribution in limiting exercise capacity with advancing age.

## 1 INTRODUCTION

Ageing leads to a progressive decline in pulmonary function as a consequence of reduced respiratory muscle strength (Tolep & Kelsen, 1993), static elastic recoil of the lungs (Knudson et al., 1977) and chest wall compliance (Johnson et al., 1994). During exercise, these changes increase the work and metabolic cost of breathing for a given level of carbon dioxide production (Johnson et al., 1994; Kipp et al., 2024; Molgat‐Seon et al., 2019). For instance, at a ventilation of 75 L/min, the total work of breathing can nearly double in older compared to younger individuals (49 vs. 90 J/min), with this difference increasing exponentially as workload and ventilation rise (Molgat‐Seon et al., 2018; Molgat‐Seon et al., 2019; Smith et al., 2018). Consequently, at high exercise intensities, similar to what is observed in young individuals (Archiza et al., 2018; Johnson et al., 1993; Romer et al., 2006), the heightened ventilatory demand and respiratory muscle work – further exacerbated by ageing – may induce substantial diaphragm and expiratory muscle fatigue, potentially leading to a significant decline in exercise capacity in older adults.

This exercise‐induced respiratory muscle fatigue does not limit the ventilatory response throughout exercise but activates a metaboreflex triggered by the stimulation of group III–IV respiratory muscle afferent nerve endings (Hill, 2000). This reflex elicits sympathoexcitation (St Croix et al., 2000) and limb vasoconstriction (Harms et al., 1997, 1998; Sheel et al., 2002), resulting in a reduced limb blood flow and oxygen delivery during exercise (Dempsey et al., 2006; Dominelli et al., 2017; Harms et al., 1997), while simultaneously redirecting 14–16% of cardiac output (CO) to the respiratory muscles (Harms et al., 1998). Accordingly, reducing the work of breathing by approximately 50–70% using a proportional assist ventilator during high intensity cycling exercise in healthy and fit individuals prevented diaphragm fatigue (Babcock et al., 2002), decreased oxygen uptake by 7%, and improved time to exhaustion by 14% (Harms et al., 1998, 2000). Additionally, when exercise was matched for duration and power output, exercise‐induced locomotor muscle fatigue was attenuated by 8% with the unloading of the respiratory muscles (Romer et al., 2006). These ergogenic effects were attributed to increased O_2_ delivery to the locomotor muscles, facilitated by the enhancement in lower limb vascular conductance (LVC) and blood flow (Harms et al., 1997, 2000). Conversely, breathing against a resistance before or during exercise to increase respiratory muscle work promotes diaphragm fatigue (Taylor & Romer, 2008; Welch et al., 2018a), and reduces LVC and blood flow, even in the presence of functional sympatholysis (Dominelli et al., 2017; Harms et al., 1997; Sheel et al., 2001). This, in turn accelerates locomotor fatigue development (Romer et al., 2006; Taylor & Romer, 2008; Wüthrich et al., 2013) and shortens time to exhaustion during constant workload cycling exercise (Mador & Acevedo, 1991; Romer et al., 2006; Taylor & Romer, 2008; Welch et al., 2018a; Wüthrich et al., 2013). These effects, which limit exercise capacity, may be further exacerbated by ageing. Indeed, older adults demonstrate a greater mean arterial pressure (MAP) response to a comparable fatiguing inspiratory task than young adults, suggesting an age‐related accentuation of the respiratory muscle metaboreflex (Leahy et al., 2023; Smith et al., 2017). While the effects of ageing on neurocirculatory adjustments remain unclear, this heightened pressure response may result from increased limb vascular responsiveness to sympathoexcitation (Koch et al., 2003) and/or impaired nitric oxide‐mediated vasodilatation (Taddei et al., 2000), both contributing to increased vascular resistance.

Although older individuals are more susceptible to respiratory muscle fatigue, its impact on exercise tolerance with ageing remains largely unexplored. Moreover, most studies in older adults have focused on sedentary individuals, whose limited aerobic capacity and lower ventilatory demands make significant respiratory muscle fatigue less likely (Mador et al., 2000). As a result, these studies may not fully capture the specific effects of ageing on the respiratory system's contribution to exercise limitations. In contrast, master athletes – typically defined as highly trained individuals over 50 years old – represent a unique model of successful ageing, minimizing the confounding effects of a sedentary lifestyle and associated deconditioning (Geard et al., 2017; Haddad et al., 2024; Lepers & Stapley, 2016). Unlike their sedentary counterparts, master athletes sustain high exercise intensities (Geard et al., 2017; Haddad et al., 2024; Lepers & Stapley, 2016), which require high levels of ventilation (>100–130 L min^−1^) and substantial respiratory effort (Haddad et al., 2024; Johnson et al., 1994, 1991; Trappe et al., 2013). Despite their high fitness levels, age‐related declines in pulmonary function persist, leading to a disproportionate increase in the respiratory muscle work required to meet ventilatory demands (Molgat‐Seon et al., 2019, 2018; Smith et al., 2018). This may further heighten susceptibility to respiratory muscle fatigue and activation of the respiratory muscle metaboreflex. However, the cardiovascular consequences of this hypothesized heightened susceptibility in ageing, along with its impact on neuromuscular fatigue and exercise performance once respiratory muscles are fatigued, remain unknown.

Accordingly, we postulated that inspiratory muscle fatigue induced by an inspiratory loading task (ILT) elicits a greater increase in resting MAP and a larger reduction in femoral blood flow in master compared to young athletes, indicative of a heightened respiratory muscle metaboreflex. We then hypothesized that pre‐existing inspiratory muscle fatigue would impair high‐intensity constant‐workload exercise performance and accelerate the development of exercise‐induced quadriceps fatigue, with a more pronounced effect in master athletes compared to young athletes. Understanding these effects is important, as they may ultimately shape exercise tolerance in ageing populations.

## 2 METHODS

### 2.1 Ethical approval

Each participant provided written informed consent before participating in the study, which was conducted according to the standards set by the latest revision of the *Declaration of Helsinki* (except for registration in a database) and approved by the national ethics committee (ID RCB: 2021‐A02808‐33).

### 2.2 Participants

Ten endurance‐trained young male athletes (YA, age: 27.4 ± 4.4 years, height: 178 ± 7 cm, body mass: 71.6 ± 9.0 kg, body fat: 13.6% ± 2.5%, peak power output (PPO): 348 ± 38 W, ${\dot{V}}_{O_{2}\max}$ = 65.9 ± 7.4 mL min^−1^ kg^−1^) and eleven endurance‐trained master male athletes (MA, age: 65.0 ± 5.1 years, height: 177 ± 5 cm, body mass: 67.9 ± 3.7 kg, body fat: 21.5 ± 3.7%, PPO: 258 ± 41 W, ${\dot{V}}_{O_{2}\max}$ = 46.5 ± 2.7 mL min^−1^ kg^−1^) participated in this study. We limited this study to males to avoid hormonal confounders from oestrogen fluctuations between sexes and across female reproductive stages, which influence cardiovascular function (Joyner & Casey, 2015). All participants were healthy, non‐smokers, free of any medication, normotensive (<139/< 89 mmHg), had standard pulmonary function (see Table 1), and had no history of cardiovascular, neurological or respiratory disease. Young and master athletes reported engaging in activities such as running, cycling and/or triathlon, were experienced in high‐intensity cycling, and trained for an average of at least 12 h and 8 h per week, respectively. Master athletes reported a history of consistent training for a minimum of 10 years. Participants were instructed to refrain from strenuous physical exercise, alcohol and caffeinated beverages for 24 h prior to each experimental session while maintaining a consistent dietary intake and training commitments throughout the period of the study. An a priori power analysis (G*Power 3.1) indicated that a minimum of six participants per group was required (repeated‐measures ANOVA, within–between interaction, α = 0.05, power = 80%, non‐sphericity correction = 1).

**TABLE 1:** Resting cardiovascular and pulmonary function.

	YA (*n* = 10)	MA (*n* = 11)	Group difference *P*
Cardiovascular function			
SBP (mmHg)	122 ± 15	118 ± 17.0	0.384
DBP (mmHg)	61 ± 7.0	67.0 ± 14	0.316
MAP (mmHg)	77 ± 8	83 ± 11.5	0.461
HR (bpm)	61 ± 10	52 ± 8	0.027
SV (mL)	71 ± 15	88 ± 37	0.188
CO (L min^−1^)	4.2 ± 0.7	4.4 ± 1.5	0.772
TPR (mmHg min L^−1^)	2.2 ± 0.8	1.8 ± 0.8	0.275
*Q̇*_L_ (mL min^−1^)	98 ± 35	134 ± 41	0.062
Pulmonary function			
FVC (L)	5.9 ± 0.8	4.9 ± 0.9	0.023
FVC (% predicted)	108.0 ± 8.7	112.4 ± 17.3	0.482
FEV_1_ (L)	4.4 ± 0.6	3.7 ± 0.8	0.031
FEV_1_ (% predicted)	98.2 ± 11.0	110.5 ± 20.5	0.106
FEV_1_/FVC (%)	75.7 ± 6.5	75.6 ± 6.5	0.983
PEF (L s^−1^)	10.1 ± 0.8	10.5 ± 1.9	0.525
FEF_25_ (L s^−1^)	8.4 ± 0.7	8.8 ± 1.8	0.553
FEF_50_ (L s^−1^)	5.3 ± 1.1	5.0 ± 1.4	0.634
FEF_75_ (L s^−1^)	2.3 ± 0.6	1.6 ± 0.6	0.008
FEF_25–75_ (L s^−1^)	4.5 ± 0.8	3.9 ± 1.1	0.228
FEF_25–75_ (% predicted)	95.9 ± 18.0	147.1 ± 39.9^*^	0.001
PI_MAX_ (cmH_2_O)	113.2 ± 15.3	102.5 ± 18.1^*^	0.164
PI_MAX_ (% predicted)	106.2 ± 14.3	119.6 ± 21.1^*^	0.106

*Note*: Cardiovascular and pulmonary function testing were performed at rest (see Methods). *Higher than Quanjer et al. (2012) predicted values (*P *< 0.05). Abbreviations: CO, cardiac output; DBP, diastolic blood pressure; FEF_25_, FEF_50_, FEF_75_, forced expiratory flow at 25%, 50% and 75% of FVC, respectively; FEF_25–75_, forced expiratory flow between 25% and 75% of FVC; FEV_1_, forced expiratory volume in 1 s; FVC, forced vital capacity; HR, heart rate; LVC, limb vascular conductance; MAP, mean arterial blood pressure; PEF, peak expiratory flow; PI_MAX_, maximal inspiratory pressure; *Q̇*_L_, limb blood flow; SBP, systolic blood pressure; SV, stroke volume; TPR, total peripheral resistance.

### 2.3 Experimental design

The study was conducted over five experimental sessions separated by a minimum of 48 h and scheduled at the same time of the day to avoid confounding biological influence of the circadian rhythm. During the first visit, participants completed cardiovascular and pulmonary function testing, followed by a maximal and graded cycling test to assess maximal oxygen consumption (${\dot{V}}_{O_{2}\max}$) and peak power output (PPO). During the second visit, participants underwent thorough familiarization with the testing procedures, starting with pulmonary and quadriceps neuromuscular function assessments at rest, followed by a 10‐min ILT at 2% (ILT_2%_, non‐fatiguing) of their maximal inspiratory pressure (PI_MAX_). Then, they performed a constant workload cycling test to exhaustion at 90% of PPO (CWT), followed by post‐exercise quadriceps neuromuscular function assessment. After approximately 20 min of rest, participants were familiarized with the ILT at 60% of their PI_MAX_ (ILT_60%_, fatiguing). During the third and fourth visit, participants completed the same tests as in the familiarization session. However, they performed the ILT at either 2% or 60% of their PI_MAX_, determined during the pulmonary function testing. The order of ILT_2%_ and ILT_60%_ was determined using a single‐blind, randomized crossover design. During the fifth visit, participants performed ILT_2%_ followed by a CWT, with power output and exercise duration matched (i.e., isotime isowork) to those of the CWT following ILT_60%_.

### 2.4 Pulmonary function testing

Following thorough familiarisation, lung function and PI_MAX_ were evaluated during each experimental visit following standard procedures (ATS/ERS Statement on Respiratory Muscle Testing, 2002). Participants performed from six to eight maximal flow volume loops to determine forced vital capacity (FVC), forced expiratory volume in 1 s (FEV_1_), FEV_1_/FVC, peak expiratory flow (PEF) and forced expiratory flow at 25–75% of vital capacity (FEF_25–75_). Spirometry manoeuvres were conducted with subjects seated in the cycling position and instantaneous visual feedback was provided for each manoeuvre. The values of the greatest maximal expiratory flow volume (MEFV) curve (e.g., greatest area under the curve, FVC and expiratory shape) were selected for analysis and compared with predicted values (Quanjer et al., 2012; Sylvester et al., 2020). To determine PI_MAX_, participants were seated in the upright position and prompted to maintain maximal inspiratory effort for 3 s from residual volume against an inspiratory resistance with a small leak to prevent glottis closure. Manoeuvres were performed with a nose clip and a mouthpiece connected to a pressure transducer (MLT844, ADInstruments, Colorado Springs, CO, USA) and repeated until three PI_MAX_ values were within a range of 5% (*n* = 5.2 ± 0.7).

### 2.5 ILT

Participants inhaled against a custom‐made flow‐resistive device allowing unrestricted exhalation. To induce inspiratory muscle fatigue, participants repeated forced inhalation to achieve a target inspiratory pressure corresponding to 60% of their PI_MAX_ during each breath (i.e., ILT_60%_). Breathing frequency and the duty cycle (i.e., ratio of inspiratory time to total breathing cycle time; *T*_i_/*T*_TOT_) were set at 15 breaths per minute and 0.7, respectively. Continuous visual feedback of mouth pressure was displayed on a monitor, while an audio metronome with distinct tones for inspiration and expiration provided feedback on breathing frequency and respiratory duty cycle. Throughout ILT, carbon dioxide end tidal partial pressure ($P_{\mathrm{ETC}O_{2}}$) was continuously monitored, and a rebreathing bag was used to prevent hypocapnia. During ILT_60%_, when participants failed to reach the targeted inspiratory pressure for three consecutive breaths despite verbal encouragement, they performed a maximal inspiratory manoeuvres. If the resulting PI_MAX_ was ≤ 85% of the resting PI_MAX_, ILT was terminated. Otherwise, participants resumed ILT_60%_. On separate sessions, participants also completed a 10‐min non‐fatiguing, sham ILT, with the inspiratory pressure target set at 2% of their PI_MAX_ (i.e., ILT_2%_).

### 2.6 Maximal and graded cycling test

To determine PPO and ${\dot{V}}_{O_{2}\max}$, participants performed a maximal and graded test on an electromagnetically braked cycle ergometer (Velotron, RacerMate, Seattle, WA, USA). The initial workload was 80 W for master athletes and 160 W for young athletes with an increase of 40 W every 3 min. Pedalling frequency was self‐selected, and exercise was stopped when participants could no longer sustain 60 rpm for more than 10 s despite verbal encouragement. To ensure that participants reached ${\dot{V}}_{O_{2}\max}$ during the maximal and graded exercise test, they performed, after a 20‐min recovery period, a constant workload exercise trial at an intensity of 110% of PPO to exhaustion. In all participants, the peak ${\dot{V}}_{O_{2}}$ from the constant workload trial was not different (*P* = 0.437) from the peak ${\dot{V}}_{O_{2}}$ from the maximal and graded exercise test, indicating that every participant reached ${\dot{V}}_{O_{2}\max}$ during the maximal and graded exercise test.

### 2.7 Constant workload test to exhaustion

Immediately after the ILT_2%_ or the ILT_60%_, participants performed a standardized 2‐min warm‐up set at 50% of PPO followed by a constant workload cycling test to exhaustion at 90% of PPO (CWT_ILT2%_ or CWT_ILT60%_, respectively), with no recovery in between. Participants were instructed to maintain a pedalling frequency of 85 rpm, and exercise was terminated when cadence fell below 5 rpm for more than 10 s despite strong verbal encouragement. No feedback about exercise time was provided during the constant load cycling test. During the fifth visit, the CWT was performed after ILT_2%_ and was stopped by experimenters when subjects reached an exercise time equal (and therefore a total amount of work) to that performed after the ILT_60%_ (CWT_ILT2%–ISO_).

### 2.8 Contractile function and voluntary activation of the quadriceps

Quadriceps muscle function was evaluated in response to maximal voluntary contraction and electrical femoral nerve stimulation. Subjects were seated on a custom‐made chair with the right knee and hip joints set at a 90° angle. The ankle was fixed to a calibrated load cell (model LC101, Omega Engineering Inc., Norwalk, CT, USA) by a non‐elastic ankle strap positioned just above the malleoli. The cathode, a self‐adhesive electrode (30 × 30 mm, Ag–AgCl, Mini‐KR; Contrôle Graphique, Brie‐Comte‐Robert, France) was placed on the femoral triangle, and the anode, a carbon‐impregnated electrode (70 × 50 mm) was placed on the gluteal fold. Both adhesive electrodes were positioned at the stimulation site leading to the highest force output and the greatest amplitude of the compound muscle action potential (*M*_MAX_) of the vastus lateralis (VL), vastus medialis (VM) and rectus femoris (RF). A square wave stimulation of 1 ms was delivered by a constant current stimulator (DS7A; Digitimer, Welwyn Garden City, UK) at an intensity set to 130% (92 ± 8 mA and 118 ± 15 mA for YA and MA, respectively) of the stimulation intensity eliciting highest quadriceps twitch and *M*_MAX_. Quadriceps neuromuscular function measurements were obtained before and after each CWT using a standardized set of contractions. In each set, participants performed a 3‐s maximal voluntary isometric contraction (MVC). During MVC, a superimposed single electrical stimulus was delivered during the peak force of the MVC to determine voluntary activation of the quadriceps (VA). Then, potentiated quadriceps twitch force evoked by single electrical stimuli of the femoral nerve (QT_SINGLE_) was elicited 3 s after MVC. Five sets, separated by 1 min, were performed pre‐exercise, with the best three measurements averaged and used as baseline values. After exercise, measurements of neuromuscular function were obtained using the same standardized set of contractions at exactly 15 s, 1 min, 2 min, 4 min, 6 min, 10 min and 15 min to capture the rapid recovery from fatigue that occurs within the first minutes after exercise termination. Quadriceps peripheral fatigue was assessed by calculating the percentage change in QT_SINGLE_ evoked force from pre‐exercise to post‐exercise. VA, an index of central fatigue, was calculated as follows: VA (%) = [1 – (QT_SINGLE, SUPERIMPOSED_/QT_SINGLE_)] × 100 (Gandevia, 2001). From QT_SINGLE_, we also determined contraction time (CT), maximal rate of force development (MRFD) and half relaxation time (HRT).

### 2.9 Electromyography

Electrical activity of the VL, the VM and the RF was recorded using wireless surface electromyography (EMG) sensors (Trigno Wireless EMG system, Delsys, Boston, MA, USA) connected to a data acquisition system (PowerLab 16/35, ADInstruments, Bella Vista, NSW, Australia). After shaving, cleaning, and abrading the skin to reduce its impedance below 3 kΩ, the sensors were positioned over the muscle belly according to the SENIAM recommendations and adjusted to maximize *M*_MAX_ amplitude. Raw EMG signals were amplified, filtered (bandwidth frequency, 0.03–1 kHz), and sampled at a rate of 2000 Hz. The EMG signal was then digitized and stored for further analysis using commercially available software (Labchart v8.1.25, ADInstruments). Each EMG burst was identified using a custom‐made analysis with MATLAB software version R2023a (MathWorks Inc., Natick, MA, USA). The root mean square (RMS) of each EMG burst was calculated during the CWT. EMG RMS values were then normalized to *M*_MAX_ and averaged over a 30 s interval.

### 2.10 Cardiovascular function

#### 2.10.1 Blood pressure

Upon arrival at the laboratory, subjects remained seated for >15 min prior to baseline cardiovascular measurements. Heart rate (HR) and calibrated beat‐to‐beat arterial blood pressure were recorded using finger pulse photoplethysmography (Finapres NOVA NC System, Finapres Medical Systems, Arnhem, Netherlands). HR, systolic blood pressure (SBP) and diastolic blood pressure (DBP) were recorded at rest and continuously during ILT. Mean arterial blood pressure (MAP) was calculated as (SBP × 2 + DBP)/3. CO, stroke volume (SV) and total peripheral resistance (TPR) were derived non‐invasively from the continuous pressure recordings (Modelflow method).

#### 2.10.2 Blood flow

Femoral artery blood velocity and artery diameter were evaluated at ∼3 cm of the bifurcation of the deep and superficial femoral artery using a Doppler ultrasound (ArtUS, Telemed Medical Systems, Lithuania) equipped with a transducer probe (L12‐5N40‐A4, Telemed) and using Echo Wave II software (version 3.4.4, Telemed). Femoral artery diameter, measured as internal diameter, and blood velocity, measured with an insonation angle <60° to the axis of the artery, were assessed at rest and every minute for 30 s during ILT using with the same probe (B‐mode imaging frequency = 12 MHz; Doppler frequency = 5 MHz. Femoral artery 2D images and Doppler spectrum were recorded as video frames for subsequent analysis. Each video frame was digitized using a custom‐made MATLAB analysis to determine femoral blood velocity, based on the pixel intensities modelled as a probability density function. Femoral cross‐sectional area (CSA) was determined from artery diameter after horizontal alignment of the lower and the upper walls. CSA was calculated as CSA = 𝜋 × *r* ^2^, with *r* being artery radius. Mean femoral blood velocity (*V*_MEAN_) was calculated as the mean blood velocity over each cardiac cycle. Femoral artery blood flow (*Q̇*_L_) was calculated as *V*_MEAN_ × CSA. LVC was calculated as: LVC = *Q̇*_L_/MAP.

#### 2.10.3 Respiratory data collection and analysis

Inspiratory and expiratory airflows were assessed with a heated pneumotachograph (3813 series, Hans Rudolph, Shawnee, KS, USA). The pneumotachograph was calibrated using a precision syringe (model 5530, Hans Rudolph), at three different flow rates (<70 L/min, ∼100–120 L/min and >150 L/min) and six volumes ranging from 0.5 to 3 L. During the entire experimental session, instantaneous flow was continuously recorded at a sampling rate of 400 Hz using a 16‐channel analog‐to‐digital data acquisition system (Powerlab 16/35, ADInstruments). Tidal volume was determined using numerical integration of the flow signal. Gas exchange was measured using a respiratory gas analyser with a spectrophotometer (Metasys TR‐M, Brainware, Toulon, France), calibrated using a reference gas (15% O_2_ and 5% CO_2_). Data analysis was conducted using a custom MATLAB script (R2023a, MathWorks Inc.).

#### 2.10.4 Perceptual responses

During the constant load cycling exercise, breathing and leg discomfort levels were assessed separately by utilizing a Borg CR‐100 every 1 min 30 s. Breathing discomfort was defined as the ‘perception of discomfort in breathing’, while leg discomfort was defined as the individual's ‘perception of discomfort in leg muscles’. For each scale, a rating of ‘0’ indicated the absence of discomfort to breath or leg discomfort, while a rating of ‘100’ indicated the most intense discomfort to breath or the highest level of leg discomfort ever experienced, respectively.

#### 2.10.5 Statistical analysis

Normality was assessed using the Shapiro–Wilk test, and homogeneity of the variance of the distributions were evaluated with Levene's test. If sphericity was violated, the Greenhouse–Geisser correction factor was applied. Descriptive characteristics, lung function data at rest, ${\dot{V}}_{O_{2}\max}$ and PPO were compared between YA and MA using an independent‐samples Student's *t*‐test. Changes in PI_MAX_ pre‐ILT versus post‐ILT, and CWT_ILT2%_ versus CWT_ILT60%_ time to exhaustion were compared using a paired *t*‐test. Effect size was assessed using Cohen's *d* and was classified as small (0.2 ≤ *d* < 0.5), medium (0.5 ≤ *d* < 0.8) or large (*d* ≥ 0.8) (Cohen, 1988). EMG‐RMS and ventilatory data during CWT were compared using three‐factor, repeated measures mixed model ANOVA with one between‐subjects variable (group: YA versus MA) and two within‐subjects variables (condition: CWT_90%–2%_ vs. CWT_ILT60%_ vs. CWT_ILT2%–ISO_; time: first to last min). Neuromuscular function was compared using three‐factor, repeated measures mixed model ANOVA with one between‐subjects variable (group: YA vs. MA) and two within‐subjects (condition: CWT_ILT2%_ vs. CWT_ILT60%_ vs. CWT_ILT2%–ISO_; time: 15 s–15 min postexercise). Cardiovascular function during ILT was compared using two‐way, repeated measures ANOVA, with one between‐subjects variable (group: YA vs. MA) and one within‐subjects variables (condition: ILT_2%_ vs. ILT_60%_). Planned pairwise comparisons were performed using Tukey post‐hoc analysis to determine where the differences resided. Effect size was assessed using partial η^2^ and was classified as small (η^2^ < 0.06), medium (0.06 ≤ η^2^ < 0.14) and large (η^2^ ≥ 0.14). All statistical analyses were completed with Jamovi (v2.4.1.0). Significance for all tests was set at *P* < 0.05. Data are expressed as means ± SD.

## 3 RESULTS

### 3.1 Participants' characteristics

Compared to YA, MA had significantly lower ${\dot{V}}_{O_{2}\max}$ (−1.64 L min^−1^ [95% CI: −2.01 to −1.27], *P* < 0.001, *d* = 4.01), PPO (−90 W [95% CI: −126 to −53], *P* < 0.001, *d* = 2.26), and higher body fat (8.2% [95% CI: 5.3 to 11.1], *P* < 0.001, *d* = 2.61), whereas height (*P* = 0.527) and weight (*P* = 0.225) were similar between groups. Both groups had similar age‐adjusted exercise capacities, with ${\dot{V}}_{O_{2}\max}$ values of 136 ± 11% and 129 ± 9% (*P* = 0.727) of the predicted age‐based value for young and master athletes, respectively (Myers et al., 2017). Results from cardiovascular, pulmonary, and metabolic functions testing at rest are presented in Table 1. Similar baseline levels of MAP, SBP, DBP, HR, CO, SV, TPR, *Q̇*_L_, LVC, ${\dot{V}}_{E}$, tidal volume (*V*_T_), breathing frequency (BF), ${\dot{V}}_{O_{2}}$, ${\dot{V}}_{CO_{2}}$, ${\dot{V}}_{E}/{\dot{V}}_{O_{2}}$ and ${\dot{V}}_{E}/{\dot{V}}_{CO_{2}}$ were found across visits in both YA and MA (all *P* > 0.090). Baseline PI_MAX_ (*p* = 0.164, *d* = 0.63), SPB (*P* = 0.384, *d* = 0.39), DBP (*P* = 0.316, *d* = 0.45), MAP (*P* = 0.461, *d* = 0.329), CO (*P* = 0.142, *d* = 0.67), TPR (*P* = 0.275, *d *= 0.49) and *Q̇*_L_ (*P* = 0.062, *d* = 0.95) were not different between YA and MA while HR was 10 bpm higher in YA (*P* = 0.027, *d* = 1.05).

### 3.2 ILTs

During ILT_60%_, PI_MAX_ decreased from baseline to task failure by 18.3 ± 9.3% (104.7 ± 14.7 vs 82.6 ± 18.1 mmHg, *P* < 0.001, *d* = 1.754) in YA and by 21.2 ± 11.3% (97.8 ± 20.9 vs. 79.7 ± 19.4 mmHg, *P* < 0.001, *d* = 1.620) in MA, with no significant difference between groups (*P* = 0.456). ILT_60%_ time to task failure was also not different (*P* = 0.378) between YA (1025 ± 212 s) and MA (899 ± 373 s). PI_MAX_ did not decrease from baseline to the end of both ILT_2%_ (YA: −1.8 ± 6.1%, *P* = 0.410; MA: −2.8 ± 4.7%, *P* = 0.259) and ILT_2%–ISO_ (YA: −1.3% ± 3.8%, *P* = 0.270; MA: −3.5% ± 9.3%, *P* = 0.312). The absence of VL, VM, and RF EMG activity during ILT_60%_, ILT_2%_ and ILT_2%–ISO_ confirmed that participants did not activate their quadriceps during the ILTs.

**FIGURE 1:**
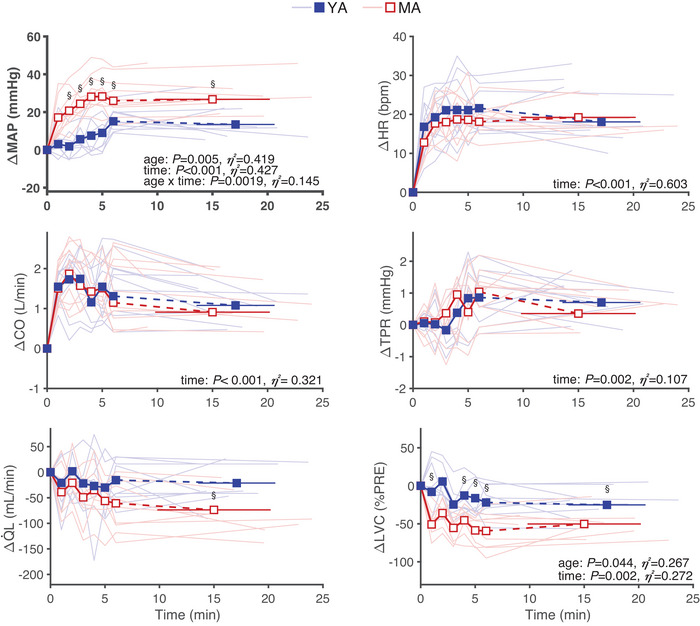
Cardiovascular response to fatiguing ILT at 60% of maximal inspiratory pressure (ILT_60%_). MAP, HR, CO, TPR, limb blood flow (*Q̇*_L_) and LVC response to ILT_60%_ in young (YA, *n* = 10, group mean shown as blue squares, individual data as shaded blue lines) and master (MA, *n* = 10, group mean shown as red open squares, individual data as shaded red lines) athletes. Note that no change in the cardiovascular indices were observed after ILT_2%_ and ILT_2% ISO_ (data not shown for figure clarity). ^§^ *P* < 0.05 between YA and MA. CO, cardiac output; HR, heart rate; ILT, inspiratory loading task; LVC, limb vascular conductance; MAP, Mean arterial pressure; TPR, total peripheral resistance.

During ILT_2%_ and ILT_2%–ISO_, there were no time, group or interaction effects for SBP, DBP, MAP, HR, CO, SV, TPR, *Q̇*_L_ and LVC (all *P* > 0.112). The cardiovascular response to ILT_60%_ is depicted in Figure 1. In both groups during ILT_60%_, there was a main effect of time, with increases observed over time in MAP (*F* = 11.16, *P* < 0.001, η*^2^* = 0.427), SBP (*F* = 6.92, *P* < 0.001, η*^2^* = 0.316), DBP (*F* = 11.42, *P* < 0.001, η*^2^* = 0.449), HR (*F* = 25.85, *P* < 0.001, η*^2^* = 0.603), CO (*F* = 12.55, *P* < 0.001, η*^2^* = 0.425) and TPR (*F* = 3.451, *P* = 0.002, η*^2^* = 0.107), and a significant decrease in LVC (*F* = 3.73, *P* = 0.002, η*^2^* = 0.272). Moreover, there was an age × condition interaction during ILT_60%_ with the increase in MAP (*F* = 10.8, *P* = 0.005, η*^2^* = 0.419), SBP (*F* = 7.47, *P* = 0.015, η*^2^* = 0.333) and DBP (*F* = 5.59, *P* = 0.033, η*^2^* = 0.285) as well as the decrease in LVC (*F* = 3.44, *P* = 0.044, η*^2^* = 0.267) being more pronounced in MA compared to YA (Figure 1). At task failure, a significant reduction in *Q̇*_L_ from baseline was found in MA only (*P* = 0.036). No significant age × condition interaction was found during ILT_60%_ for HR (*F* = 0.519, *P* = 0.481, η*^2^* = 0.030), CO (*F* = 0.01, *P* = 0.943, η*^2^* = 0.000), SV (*F* = 0.458, *P *= 0.507, η*^2^* = 0.025) or TPR (*F* = 0.01, *P* = 0.915, η*^2^* = 0.000).

### 3.3 Exercise performance and EMG

**FIGURE 2:**
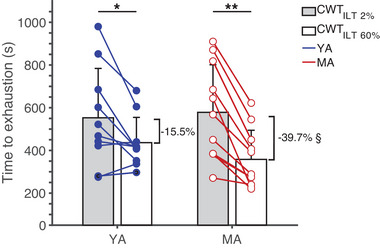
Time to exhaustion during the CWT. The constant workload cycling test was performed after the ILT at 60% of maximal inspiratory pressure (CWT_ILT60%_) and, on separate days, after a sham condition (CWT_ILT2%_). Group mean (bars), and individual (circles and lines) data are shown for each condition, with young athletes (YA, *n* = 10) represented by blue filled circles and master athletes (MA, *n* = 11) by red open circles. Power output was identical between CWT_ILT60%_ and CWT_ILT2%_. ^*^ *P* < 0.05 between CWT_ILT60%_ and CWT_ILT2%_; ^**^ *P* < 0.001 between CWT_ILT60%_ and CWT_ILT2%_; ^§^ *P* < 0.05 between YA and MA. CWT, constant workload cycling tests; ILT, inspiratory loading task.

As shown in Figure 2, there was no significant main effect of age on time to exhaustion during cycling exercise (*F* = 0.005, *P* = 0.942, η*^2^* = 0.000). However, a significant main effect of condition was observed (*F* = 34.04, *P* < 0.001, η*^2^* = 0.642), with participants having a shorter time to exhaustion during the CWT_ILT60%_ (MA: 5.1 ± 2.3 min; YA: 6.4 ± 2.0 min) compared to the CWT_ILT2%_ (MA: 8.8 ± 2.9 min; YA: 8.4 ± 3.1 min) (*P* < 0.001). There was also a significant age × condition interaction (*F* = 4.69, *P* = 0.041, η*^2^* = 0.207), indicating that MA experienced a 2.6 times greater reduction in time to exhaustion during CWT_ILT60%_ compared to YA.

**FIGURE 3:**
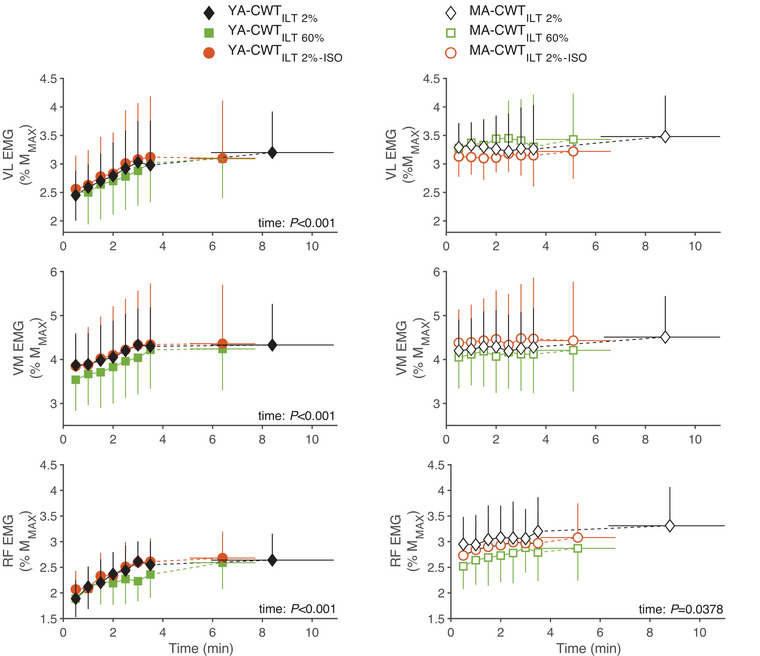
Quadriceps muscle activation during the CWT. The RMS of the VL, VM and RF EMGs measured during exercise in young (YA, *n* = 10, black, left panel) and master (MA, *n* = 10, white, right panel) athletes was normalized to the *M*_MAX_ recorded during the pre‐exercise MVC of the quadriceps (%MVC). CWT was performed following an ILT at 60% (CWT_ILT60%_) and 2% (i.e., sham conditions CWT_ILT2%_ and CWT_ILT2%–ISO_) of each participant's maximal inspiratory pressure. CWT_ILT60%_ and CWT_ILT2%_ were performed to exhaustion. CWT_ILT2%–ISO_ was interrupted at the time equal to that performed during CWT_ILT60%_. CWT, constant workload cycling tests; ILT, inspiratory loading task; MVC, maximal voluntary isometric contraction; RF, rectus femoris; RMS, root mean square; VL, vastus lateralis; VM, vastus medialis.

In both groups, there was no main effect of condition on VL, VM or RF normalized RMS EMG (*F* = 0.242–1.294, all *P* > 0.124, η*^2^* = 0.077 – 0.105) (Figure 3). In YA, a significant increase over time was observed for VL‐EMG (*F* = 3.799, *P* < 0.001, η*^2^* = 0.592), VM‐EMG (*F* = 5.756, *P* < 0.001, η*^2^* = 0.650) and RF‐EMG (*F* = 3.154, *P* < 0.001, η*^2^* = 0.403). In MA, a significant increase over time was observed for RF‐EMG (*F* = 2.070, *P* = 0.0378, η*^2^* = 0.250).

### 3.4 Neuromuscular fatigue

Neuromuscular fatigue variables at baseline and at exercise termination are presented in Table 2 and Figure 3. Across conditions, similar baseline levels of MVC, QT_SINGLE_, VA, MRFD, HRT, VL *M*_MAX_, VM *M*_MAX_, and RF *M*_MAX_ were found in both YA (*P* > 0.457) and MA (*P* > 0.236).

**TABLE 2:** Neuromuscular function indices at baseline and at exercise termination.

		CWT_ILT2%_		CWT_ILT60%_		CWT_ILT2%–ISO_		*P*		
		YA (*n* = 10)	MA (*n* = 10)	YA (*n* = 10)	MA (*n* = 10)	YA (*n* = 10)	MA (*n* = 10)	Main effect of age	Main effect of condition	Interaction effect
MVC (N)	Pre	609 ± 100	447 ± 73^*^	597 ± 114	441 ± 82^*^	621 ± 118	427 ± 95^*^	<0.001	0.237	0.847
	Post‐15 s	486 ± 118^**^	378 ± 77^*,**^	497 ± 117^**^	379 ± 86^*,**^	516 ± 144^**^	385 ± 90^*,**^	0.028	0.044	0.162
QT_SINGLE_ (N)	Pre	194 ± 30	120 ± 45^*^	197 ± 31	142 ± 38^*^	188 ± 17	136 ± 34^*^	0.008	0.163	0.709
	Post‐15 s	140 ± 33^**^	113 ± 41^*,**^	133 ± 36^**^	113 ± 44^*,**^	133 ± 45^**^	122 ± 41^**^	0.579	0.120	0.298
VA (%)	Pre	93 ± 3	95 ± 3	93 ± 3	95 ± 4	94 ± 5	96 ± 3	0.345	0.214	0.114
	Post‐15 s	91 ± 5	91 ± 6^**^	90 ± 8	92 ± 6	93 ± 5	96 ± 5	0.579	0.040	0.297
CT (ms)	Pre	53 ± 24	78 ± 7^*^	52 ± 21	77 ± 8^*^	59 ± 22	70 ± 16	0.004	0.573	0.006
	Post‐15 s	48 ± 16	59 ± 20^**^	48 ± 17	59 ± 19^**^	49 ± 18	53 ± 18^**^	0.107	0.243	0.194
MRFD (N ms^−1^)	Pre	6.7 ± 2.3	4.3 ± 1.5^*^	6.8 ± 3.2	4.5 ± 2.2^*^	6.5 ± 1.4	4.0 ± 1.4^*^	0.012	0.030	0.179
	Post‐15 s	3.6 ± 2.5^**^	3.2 ± 1.8^**^	4.4 ± 2.6^**^	2.8 ± 1.4^*,**^	4.4 ± 2.4^**^	3.0 ± 1.6	0.244	0.402	0.026
HRT (ms)	Pre	90 ± 15	117 ± 42	90 ± 15	92 ± 32	85 ± 16	112 ± 33^*^	0.080	0.660	0.824
	Post‐15 s	87 ± 25	111 ± 35^*^	108 ± 33^**^	130 ± 46^*,**^	91 ± 21	114 ± 51^*^	0.022	0.240	0.714
VL *M*_MAX_ (mV)	Pre	8.0 ± 1.3	6.1 ± 2.6^*^	7.9 ± 2.0	6.3 ± 2.9^*^	7.5 ± 1.7	5.9 ± 3.0^*^	<0.001	0.521	0.321
	Post‐15 s	7.8 ± 1.4	6.2 ± 2.9^*^	8.0 ± 2.3	6.0 ± 3.0^*^	8.0 ± 2.0	6.3 ± 2.7^*^	<0.001	0.125	0.356
VM *M*_MAX_ (mV)	Pre	10.4 ± 1.2	9.1 ± 2.3^*^	11.0 ± 0.2	9.1 ± 2.5^*^	10.4 ± 0.7	9.1 ± 3.2^*^	0.025	0.231	0.198
	Post‐15 s	10.9 ± 0.3	9.0 ± 2.6	10.2 ± 1.5	9.6 ± 2.0	10.5 ± 0.5	10.3 ± 1.6	0.080	0.532	0.215
RF *M*_MAX_ (mV)	Pre	3.8 ± 1.5	3.1 ± 1.0	3.6 ± 1.3	3.7 ± 1.3	3.2 ± 1.0	3.4 ± 1.4	0.211	0.471	0.288
	Post‐15 s	3.1 ± 1.0	3.9 ± 1.6	3.2 ± 1.0	3.5 ± 1.0	3.5 ± 1.0	3.3 ± 1.1	0.126	0.199	0.281

^*^ *P* < 0.05 between YA and MA; ^**^ *P* < 0.05, between pre‐ and post‐exercise. Abbreviations: CT, contraction time; CWT_ILT2%_ and CWT_ILT60%_, constant workload trial at 90% of peak power after 2% and 60% ILT, respectively; CWT_ILT2%–ISO_, constant workload trial at 90% of peak power after 2% ILT, terminated when time and work matched those of CWT_ILT60%_; HRT, half relaxation time; MA, master athletes; MRFD, maximal rate of force development; MVC, maximal voluntary contraction; QT_SINGLE_, potentiated quadriceps twitch evoked with a single supramaximal electrical stimulation; VA, voluntary activation of the quadriceps; VL *M*_MAX_, VM *M*_MAX_, RF *M*_MAX_, maximal amplitude of the compound muscle action potential for the VL, the VM and the RF, respectively; YA, young athletes.

YA had greater baseline MVC (Δ = 174 ± 41 N, *P* < 0.001), QT_SINGLE_ (Δ = 47.6 ± 16 N, *P* = 0.008) and MRFD (Δ = 1.8 ± 0.6 N ms^−1^, *P* = 0.012), and lower CT (Δ = 23 ± 7 ms, *P* = 0.004) than MA.

At the end of exercise (i.e., 15 s), both groups showed significant exercise‐induced reductions in MVC, QT_SINGLE_ and MRFD (all *P* < 0.001) across all conditions. In both groups, there was no significant difference in any neuromuscular variables between CWT_ILT60%_ and CWT_ILT2%_ (*P* > 0.055). In MA, MVC (Δ MVC_MA_ = 7.3% ± 6.5%) and QT_SINGLE_ (Δ QT_SINGLE‐MA_ = 10.8% ± 10.9%) decreased more after CWT_ILT60%_ than after CWT_ILT2%–ISO_ (*P* < 0.039)_._ In YA, no significant differences were found in MVC (Δ MVC_YA_ = 2.2% ± 7.6%) and QT_SINGLE_ (Δ QT_SINGLE_‐_YA_ = 3.2% ± 7.4%) (both *P* > 0.697) between CWT_ILT60%_ and CWT_ILT2%–ISO_. At 15 s post‐exercise, the decrease in QT_SINGLE_ was lower in MA than in YA across all conditions, and the decrease in MVC was lower in MA than in YA for CWT_ILT60%_ and CWT_ILT2%–ISO_. Significant reductions in VA from baseline were observed 15 s post‐CWT_ILT2%_ in MA, and 2 min post‐CWT_ILT60%_ and 4 min post‐CWT_ILT2%_ in YA. VA was lower after CWT_ILT60%_ compared to CWT_ILT2%–ISO_ in MA (*P* = 0.047). There was no main effect of age, condition or age × condition interaction for VL‐RMS_MVC_, VM‐RMS_MVC_ and RF‐RMS_MVC_ from baseline to post‐exercise (*F* = 0.129–0.178, all *P* > 0.094, η*^2^* = 0.090–0.099).

**FIGURE 4:**
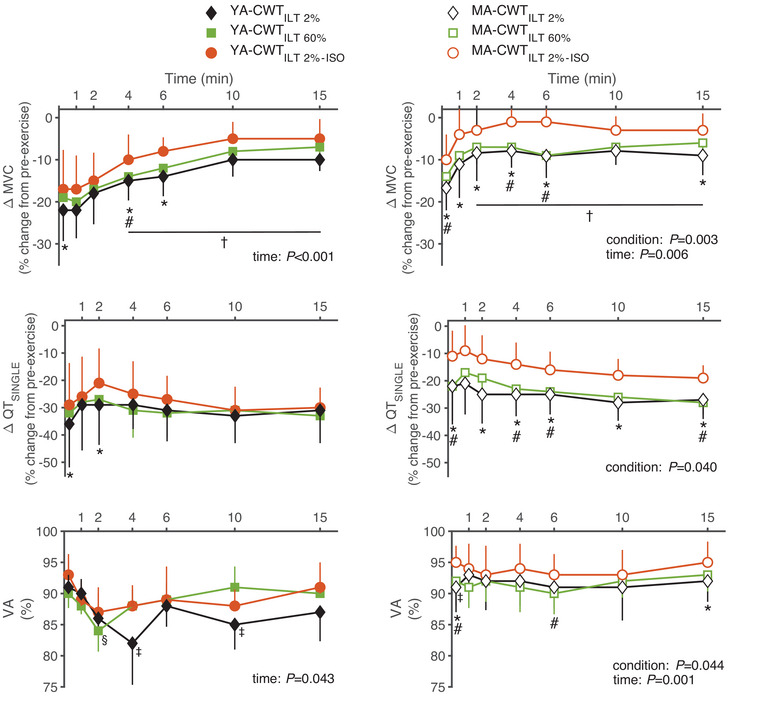
Exercise‐induced neuromuscular fatigue. Pre‐ to post‐exercise difference in MVC, in potentiated twitch force evoked by a single electrical stimulation of the femoral nerve (QT_SINGLE_) and in VA in young (YA, *n* = 10, filled, left panel) and master (MA, *n* = 10, open, right panel) athletes. The CWT was performed following and ILT at 60% (CWT_ILT60%_) and 2% (i.e., sham conditions CWT_ILT2%_ and CWT_ILT2%–ISO_) of each participant's maximal inspiratory pressure. CWT_ILT60%_ and CWT_ILT2%_ were performed to exhaustion. CWT_ILT2%–ISO_ was interrupted at the time equal to that performed during CWT_ILT60%_. ^*^ *P* < 0.05 between CWT_ILT2%_ and CWT_ILT2%–ISO_; ^#^ *P* < 0.05 between CWT_ILT60%_ and CWT_ILT2%–ISO_; ^†^ *P* < 0.05 between 15 s and the corresponding point; ^‡^ *P *< 0.05 between VA baseline and the corresponding point in CWT_ILT2%;_^§^ *P *< 0.05 between VA baseline and the corresponding point in CWT_ILT60%_. CWT, constant workload cycling tests; ILT, inspiratory loading task; MVC, maximal voluntary contractions; VA, voluntary activation.

Data showing recovery from exercise‐induced neuromuscular fatigue are presented in Figure 4. In both groups, a significant (*F* = 8.343, *P* = 0.042, η*^2^ *= 0.216) but incomplete MVC recovery toward baseline values was observed. In contrast, no recovery from peripheral fatigue was found during the 15 min recovery period, as evidenced by the lack of a time effect for QT_SINGLE_ (*F* = 1.387, *P* = 0.270, η^2^ = 0.112) and MRFD (*F* = 1.533, *P* = 0.176, η*^2^* = 0.087). Similar to observations at 15 s post‐exercise, the exercise‐induced reductions in MVC and QT_SINGLE_ were less throughout most of the recovery period after CWT_ILT2%–ISO_ compared to CWT_ILT60_ and CWT_ILT2%_ in MA.

### 3.5 Ventilatory, HR, metabolic data and perceptual responses

**FIGURE 5:**
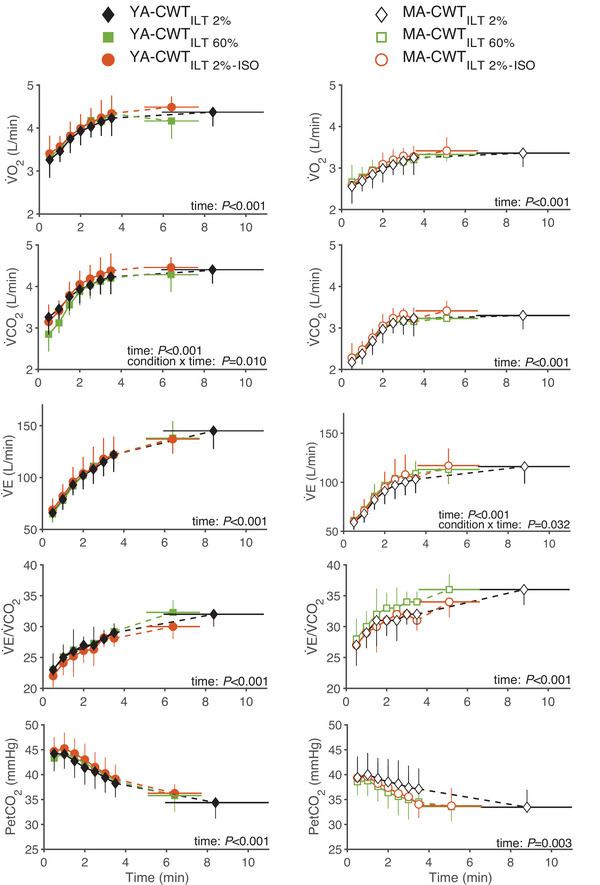
Metabolic and ventilatory response to the CWT. Changes in O_2_ uptake (${\dot{V}}_{O_{2}}$), CO_2_ production (${\dot{V}}_{CO_{2}}$), minute ventilation (${\dot{V}}_{E}$), ventilatory equivalent for CO_2_ (${\dot{V}}_{E}/{\dot{V}}_{CO_{2}}$) and partial pressure of end tidal CO_2_ ($P_{\mathrm{ETC}O_{2}}$) during CWT in young (YA, *n* = 10, filled symbols, left panel) and master (MA, *n* = 11, open symbols, right panel) athletes. CWT was performed following and ILT at 60% (CWT_ILT60%_) and 2% (i.e., sham conditions CWT_ILT2%_ and CWT_ILT2%–ISO_) of each participant's maximal inspiratory pressure. CWT_ILT60%_ and CWT_ILT2%_ were performed to exhaustion. CWT_ILT2%–ISO_ was interrupted at the time equal to that performed during CWT_ILT60%_. CWT, constant workload cycling tests; ILT, inspiratory loading task.

Ventilatory and metabolic data during CWT are depicted in Figure 5. ${\dot{V}}_{E}$ (*F* = 152.592, *P* < 0.001, η*^2 ^* = 0.921), ${\dot{V}}_{O_{2}}$ (*F* = 38.40, *P* < 0.001, η*^2^* = 0.761), ${\dot{V}}_{CO_{2}}$ (*F* = 40.57, *P* < 0.001, η*^2^* = 0.851), ${\dot{V}}_{E}/{\dot{V}}_{CO_{2}}$ (*F* = 28.60, *P* < 0.001, η*^2^* = 0.688), BF (*F* = 14.50, *P* = 0.020, η*^2^* = 0.308) and HR (*F* = 370.63, *P* < 0.001, η*^2^* = 0.970) increased from the beginning to the end of exercise, while $P_{\mathrm{ETC}O_{2}}$ (*F* = 22.09, *P* < 0.001, η*^2^* = 0.453) decreased from the first minute to the end of exercise. *V*_T_ increased (*F* = 32.12, *P* < 0.001, η*^2^* = 0.682) from the beginning of exercise until the second minute and then plateaued or tended to slightly decrease. ${\dot{V}}_{E}$, ${\dot{V}}_{O_{2}}$, ${\dot{V}}_{CO_{2}}$ and HR were significantly higher in YA than in MA (*F* = 5.336–8.650, all *P* < 0.005, η*^2^* = 0.639–0.720). For $S_{pO_{2}}$, there was no main effect of condition nor main effect of age (*F* = 0.182–0.196, both *P *> 0.676, η*^2^* = 0.016–0.018). However, there was a main effect of time (*F* = 14.470, *P* < 0.001, η*^2^* = 0.568), with $S_{pO_{2}}$ decreasing from 99% to 96%, from the first to the last minute of CWT. At the end of exercise, capillary blood lactate concentration ([La]_b_) was not different across all conditions (*F* = 1.657, *P* = 0.207, η^2 ^= 0.094) but was higher in YA compared to MA (YA: 12.9 ± 3.5 vs. MA: 7.9 ± 2.4 mmol L^−1^, *P* = 0.002).

**FIGURE 6:**
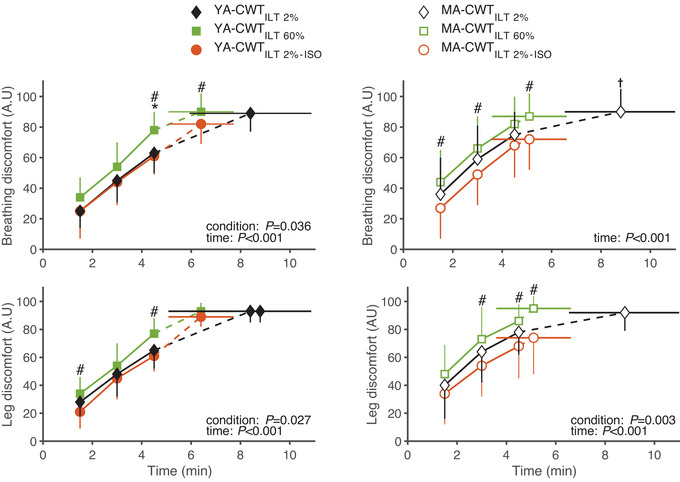
Ratings of perceived breathing and leg discomfort during the CWT. Perceptual responses to CWT, in young (YA, *n* = 10, filled, left panels) and master (MA, *n* = 11, open, right panel) athletes. CWT was performed following and ILT at 60% (CWT_ILT60%_) and 2% (i.e., sham conditions CWT_ILT2%_ and CWT_ILT2%–ISO_) of each participant's maximal inspiratory pressure. CWT_ILT60%_ and CWT_ILT2%_ were performed to exhaustion. CWT_ILT2%–ISO_ was interrupted at the time equal to that performed during CWT_ILT60%_. ^*^ *P* < 0.05 between CWT_ILT2%_ and CWT_ILT60%_; ^#^ *P* < 0.05 between CWT_ILT2%–ISO_ and CWT_ILT60%_; ^†^ *P* < 0.05 between CWT_ILT2%_ and CWT_ILT2%–ISO_. CWT, constant workload cycling tests; ILT, inspiratory loading task.

Perceptual responses during CWT are shown in Figure 6. In both groups, breathing discomfort (*F* = 196.4, *P* < 0.001, η*^2^* = 0.942) and leg discomfort (*F* = 110.505, *P* < 0.001, η*^2^* = 0.880) increased gradually over time across all conditions. No differences in perceptual responses were found between CWT_ILT2%_ and CWT_ILT2%–ISO_. Breathing discomfort (*F* = 8.183, *P* = 0.002, η*^2^* = 0.405) was, however, higher during CWT_ILT60%_ than during CWT_ILT2%_ and CWT_ILT2%–ISO_. Leg discomfort was higher during CWT_ILT60%_ than during CWT_ILT2%–ISO_ (*F* = 11.208, *P* < 0.001, η*^2^* = 0.428) and during the first part of exercise in MA compared to YA (*F* = 4.370, *P* = 0.009, η*^2^* = 0.226). Finally, the difference in leg discomfort at the end of exercise between CWT_ILT60%_ and CWT_ILT 2%–ISO_ was more pronounced in MA compared to YA (*P* = 0.048).

## 4 DISCUSSION

Our research investigated how healthy ageing influences the adverse effects of respiratory muscle fatigue on cardiovascular function, as well as on locomotor muscle fatigue and exercise performance during a subsequent high intensity cycling constant workload trial. Master athletes showed a heightened respiratory muscle metaboreflex compared to young athletes, as evidenced by an increased MAP, greater reduction in vascular conductance and reduced blood flow in response to fatiguing inspiratory muscle loading. During the subsequent constant workload trial, these effects substantially impaired time to exhaustion in both groups, with these impairments being more pronounced in master athletes. When exercise duration and work were matched, fatiguing inspiratory muscle loading before exercise (i.e., CWT_ILT60%_) exacerbated exercise‐induced reduction in maximal voluntary contraction and potentiated twitch compared to sham (i.e., CWT_ILT2% ISO_), with this effect being particularly pronounced in master athletes. This finding highlights that ageing exacerbates the respiratory muscle metaboreflex and accentuates the impact of pre‐existing respiratory muscle fatigue on locomotor muscle fatigue during exercise, further compromising the ability of older athletes to sustain high‐intensity efforts. It underscores the increasingly limiting role of respiratory muscle fatigue in exercise capacity and tolerance with ageing.

### 4.1 Cardiovascular response to respiratory muscle fatigue

To enable the comparison with findings from untrained young and old participants, we used an ILT similar to those used in previous studies (Geary et al., 2019; Peters et al., 2017; Sheel et al., 2001; Welch et al., 2018a, 2018b). This protocol effectively reduces transdiaphragmatic twitch pressure, indicative of inspiratory muscle fatigue (Geary et al., 2019; Peters et al., 2017; Welch et al., 2018a, 2018b), and activates the respiratory muscle metaboreflex (Sheel et al., 2001, 2002; St Croix et al., 2000). Both groups showed a significant rise in MAP and an 18–21% reduction in PI_MAX_ following ILT_60%_, with these responses absent in the sham condition (i.e., ILT_2%_). These findings suggest activation of the respiratory metaboreflex and inspiratory muscle fatigue, respectively. Moreover, master athletes showed a more pronounced pressure response to ILT_60%_ than young athletes, resembling the magnitude observed in untrained old individuals (Leahy et al., 2023). This indicates that the heightened respiratory muscle metaboreflex with ageing is likely an intrinsic characteristic of ageing, rather than a consequence of reduced physical activity. Our pressure findings are also consistent with those of studies on the exercise pressor reflex, which showed an age‐related exacerbation of arterial pressure responses during fatiguing exercise. For example, D'Souza et al. (2023) reported a greater MAP in older males compared to young males at peak handgrip exercise (123 ± 15 vs. 98 ± 6 mmHg, respectively), while Hasegawa et al. (2021) observed a higher SPB response in older males compared to younger males (135 ± 4 vs. 124 ± 2 mmHg, respectively) during rhythmic handgrip exercise (Hasegawa et al., 2021). In contrast to our findings and those from Leahy et al. (2023), Smith et al. (2017) found no significant effect of age on MAP and limb vascular resistance in males during inspiratory muscle loading. This discrepancy may be due to differences in experimental protocols, including earlier termination of the task and a smaller MAP increase in their study. Therefore, the effect of age on the respiratory metaboreflex may become more evident during more intense breathing tasks conducted to failure.

The increased MAP observed in master athletes was associated with a marked decrease in LVC compared to young athletes, while CO and HR increases were similar between the groups. The reduction in femoral blood flow during fatiguing respiratory muscle loading observed in MA is thus likely driven by increased vascular resistance rather than changes in central haemodynamics (Milia et al., 2015). This interpretation is supported by evidence showing that fatiguing inspiratory muscle work increases muscle sympathetic nerve activity (MSNA) by 77% compared to control (Sheel et al., 2001; St Croix et al., 2000). Accordingly, the elevated MAP and reduced vascular conductance in master athletes during ILT_60%_ are likely mediated by enhanced sympathetically meditated vasoconstriction. Conversely, the contribution of endothelial dysfunction – an age‐related condition that impairs peripheral vasodilatation and increases peripheral resistance (Milia et al., 2015) – to the heightened pressor response with age seems limited. The increase in MAP during ILT_60%_ was similar between our master athletes and untrained old individuals (Leahy et al., 2023), despite chronic endurance training effectively mitigating age‐related declines in nitric oxide availability and endothelial function (Taddei et al., 2000).

Finally, in contrast to the findings of Leahy et al. (2023), which showed lower resting PI_MAX_ and greater reduction in time to task failure with age during fatiguing respiratory muscle loading in untrained individuals, we found no such differences between master and young athletes. This suggests that lifelong endurance training may mitigate the typical age‐related decline in respiratory muscle strength and resistance to fatigue (Summerhill et al., 2007).

### 4.2 Consequences of pre‐existing respiratory muscle fatigue on exercise performance

Pre‐existing respiratory muscle fatigue led to a 15.5% reduction in time to exhaustion in young athletes, which is consistent with the previously reported 14–23% reduction in exercise performance following fatiguing respiratory muscle loading in young untrained and moderately trained adults (Mador & Acevedo, 1991; Welch et al., 2018a; Wüthrich et al., 2013). They contrast, however, with results showing no performance decline during constant workload cycling following respiratory muscle loading via isocapnic hyperpnoea (Dodd et al., 1989; Spengler et al., 2000). Since transdiaphragmatic or mouth pressures were not reported in these studies, it is possible that their loading protocol failed to induce sufficient respiratory muscle fatigue to impair performance.

Our unique data in older, endurance trained individuals show that the fatiguing ILT impaired exercise performance to a much greater extent (i.e., ∼40%) compared to their young counterparts, demonstrating that ageing accentuates the adverse effects of the respiratory muscle fatigue on exercise performance and tolerance. Why did fatiguing inspiratory muscle loading limit time to exhaustion more in master athletes? Because there were no significant differences in ${\dot{V}}_{E}/{\dot{V}}_{O_{2}}$, ${\dot{V}}_{E}/{\dot{V}}_{CO_{2}}$, $P_{\mathrm{ETC}O_{2}}$ or $S_{pO_{2}}$ between the pre‐existing respiratory muscle fatigue and sham condition in either group, we do not attribute this finding to inadequate ventilatory responses or reduced arterial oxygenation. We propose that the reduced LVC and blood flow observed at the end of the fatiguing ILT, particularly evident in master athletes, persisted during the subsequent cycling exercise, which began immediately after the inspiratory task. This is supported by data from Katayama et al. (2012), which showed that inspiratory resistive breathing during leg cycling led to a time‐dependent increase in MSNA burst frequency, suggesting that diaphragm fatigue increases sympathetic vasoconstrictor outflow during exercise. Importantly, this increase in MSNA persisted for at least 5 min after the completion of the respiratory muscle loading task (Katayama et al., 2012). Additionally, in a dog model, stimulating diaphragm metaboreceptors with local lactic acid infusion during exercise decreased vascular conductance, and reduced hindlimb muscle blood flow (Rodman et al., 2003), demonstrating that reflex vasoconstriction can override exercise‐induced vasodilatation. Ageing may further influence the cardiovascular response to respiratory muscle fatigue during exercise through various adaptations independent of increased sympathetic tone. Specifically, data from healthy but untrained older men suggest that vasoconstrictor responsiveness to acute sympathetic stimulation during exercise (i.e., hand cold pressor test and α‐adrenergic receptor stimulation) is enhanced (Koch et al., 2003). At the same time, functional sympatholysis is impaired in the legs (Koch et al., 2003) and the forearm (Dinenno et al., 2005) during dynamic exercise, indicating impaired vasodilatory control during exercise. Ageing is also associated with structural changes to the respiratory system, including a loss of elastic recoil of the lungs and a stiffening of the chest wall (Johnson et al., 1991). As a result, master athletes showed a 15–20% reduction in FVC and FEV_1_ compared to young athletes (Table 1). These structural changes, which are only minimally mitigated by endurance training (McClaran et al., 1995), increase pulmonary resistance and respiratory muscle work for a given ventilation during exercise. Consequently, the metabolic cost of contractions and the blood flow demand of the respiratory muscles are heightened (Kipp et al., 2024). This could exacerbate the adverse effects of the respiratory muscle metaboreflex on blood flow to the locomotor muscles, compromising O_2_ delivery, accelerating the accumulation of intramuscular metabolites and precipitating the development of neuromuscular fatigue.

### 4.3 Consequences of pre‐existing respiratory muscle fatigue on quadriceps fatigue

Consistent with this proposal, our fatigue data show that the exercise‐induced reduction in MVC was Δ7.3% greater in master athletes following CWT_ILT60%_ compared to CWT_ILT2%ISO_, despite both groups completing a similar exercise duration and amount of work. The minimal and inconsistent exercise‐induced reduction in VA, combined with no pre‐ to post‐exercise changes in RMS amplitude during MVC, suggests that central fatigue had little influence on the MVC differences between conditions and groups. Instead, the reduction in the ability to voluntary generate force can be mainly attributed to peripheral fatigue mechanisms, as evidenced by the Δ10.8% reduction in QT_SINGLE_ during CWT_ILT60%_ compared to CWT_ILT2%–ISO_ in MA. The absence of exercise‐induced reduction in M‐wave amplitude indicates that peripheral fatigue was not determined by reduced membrane excitability. Instead, the combination of reduced MRFD and lengthened CT and HRT suggests that alterations in excitation–contraction coupling, driven by intramuscular metabolite accumulation (Allen et al., 2008), are more likely responsible for reductions in QT_SINGLE_.

The effects of pre‐existing respiratory muscle fatigue on neuromuscular fatigue were primarily observed in master athletes. In contrast, young athletes showed no significant reductions in most of the indices of exercise‐induced neuromuscular fatigue following ILT_60%_ compared to ILT_2%–ISO_. Our findings differ from those of previous studies, which reported greater quadriceps muscle fatigue after cycling with prior inspiratory (Wüthrich et al., 2013) or expiratory (Taylor & Romer, 2008) muscle fatigue compared to without. This discrepancy might be explained by the higher fitness level of our participants (${\dot{V}}_{O_{2}\max}$ > ∼9–12 mL min^−1^ kg^−1^), suggesting that endurance training may mitigate the adverse effects of respiratory muscle fatigue on cardiovascular function (Callegaro et al., 2011) and neuromuscular fatigue development. This is consistent with our findings showing a minimal effect of fatiguing inspiratory muscle loading on leg vascular conductance and blood flow in young athletes. Inspiratory muscle loading prior to exercise, however, increased leg discomfort and dyspnoea during CWT_ILT60%_, likely triggering a negative feedback mechanism that contributed to premature exercise failure and a decline in performance (Dempsey et al., 2008).

There was no effect of prior respiratory muscle fatigue on quadriceps EMG relative to *M*_MAX_ during CWT, suggesting similar muscle activity across conditions. Consistent with previous findings in untrained young and old individuals (Ferri et al., 2007), master athletes showed a lower rate of EMG increase during exercise than young athletes, with VL and VM‐EMG remaining unchanged throughout exercise. This could be attributed to a higher proportion of type I motor units, which have a lower depolarization threshold (Freund et al., 1975) and are more prevalent in older individuals who train regularly for endurance events (Trappe et al., 1995), leading to near‐maximal motor unit recruitment from the onset of exercise.

In this study, neuromuscular function was assessed from 15 s to 15 min post‐exercise to provide an accurate evaluation of short‐term recovery from neuromuscular fatigue. Notably, both groups demonstrated no recovery from peripheral fatigue, as evidenced by the lack of change in QT_SINGLE_ throughout the recovery period. This absence of recovery might be a characteristic of highly trained endurance athletes (Ducrocq et al., 2021). The early phase of peripheral fatigue recovery following severe‐intensity locomotor exercise is primarily driven by the clearance of intramuscular metabolites associated with phosphocreatine hydrolysis and ATP depletion (Baker et al., 1993; Bogdanis et al., 1995; Miller et al., 1987). Our findings thus indirectly suggest that metabolite buildup during exercise was constrained in our participants, especially in MA, who showed less peripheral fatigue and lower blood lactate concentration post‐exercise compared to younger athletes. Chronic endurance training induces long‐term intramuscular adaptations that may reduce metabolite accumulation or decrease myofibrillar sensitivity to these metabolites during exercise (Costill et al., 1976; Hug et al., 2005; Place et al., 2015). Such adaptations include a higher proportion of slow‐twitch fatigue‐resistant muscle fibres (Hamada et al., 2003), enhanced reliance on oxidative metabolism (Hug et al., 2005), increased oxidative enzymes activity (Costill et al., 1976) and/or reduced exercise‐induced fragmentation of ryanodine receptors (Place et al., 2015).

### 4.4 Limitations and technical considerations

The absence of invasive oesophageal pressure measurements limited direct assessment of respiratory muscle pressure‐generating capacity. Instead, we used maximal inspiratory pressures, a valid non‐invasive approach (ATS/ERS Statement on Respiratory Muscle Testing, 2002), to estimate inspiratory muscle fatigue. While this method has limitations, such as reliance on participant motivation and volitional effort, as well as the potential influence from accessory muscles, baseline PI_MAX_ was determined following thorough familiarization, using visual feedback and with variations of <5% between consecutive measurements and between sessions. Importantly, reductions in PI_MAX_ after ILT_60%_ are consistent with results from previous studies using similar inspiratory muscle loading tasks, where reductions of transdiaphragmatic pressure indicated diaphragmatic fatigue (Peters et al., 2017; Welch et al., 2018b). The absence of direct MSNA measurements limits our ability to fully assess the role of ageing in respiratory muscle fatigue‐mediated sympathetic nervous activity. To address this limitation, the ILT_60%_ protocol was designed to replicate conditions shown to induce time‐dependent increases in limb MSNA (St Croix et al., 2000). Due to the high intensity nature of exercise (i.e., 90% of PPO), Doppler ultrasound could not be used to measure limb blood flow during cycling, limiting our insight into whether reduced leg blood flow with respiratory muscle fatigue in master athletes persisted during exercise. Additionally, including a sedentary young and older group would have provided further insight into how ageing and sedentary behaviour interact to influence the effects of pre‐existing respiratory muscle fatigue on locomotor muscle fatigue and exercise capacity. Finally, investigating the role of biological sex in physiological responses to respiratory muscle fatigue is an important question that warrants a dedicated study.

### 4.5 Conclusion

Ageing exacerbates the cardiovascular, neuromuscular and performance consequences of respiratory muscle fatigue. In master athletes, this resulted in higher MAP, greater reductions in LVC and blood flow, and accelerated peripheral fatigue during subsequent cycling exercise, leading to more severe performance impairments compared to young athletes. These findings highlight how ageing heightens the impact of respiratory muscle fatigue on cardiovascular function, exacerbates neuromuscular fatigue and decreases exercise performance, suggesting an increasing role of the respiratory system in constraining exercise capacity with age.

## AUTHOR CONTRIBUTIONS

All authors conceptualized and designed the study. Valentin Mons and Colin Lavigne collected the data. All authors contributed to the interpretation of data and results, and revisions of intellectual content. Valentin Mons drafted the first manuscript, and all authors critically revised the manuscript. All authors have read and approved the final version of this manuscript and agree to be accountable for all aspects of the work in ensuring that questions related to the accuracy or integrity of any part of the work are appropriately investigated and resolved. All persons designated as authors qualify for authorship, and all those who qualify for authorship are listed.

## CONFLICT OF INTEREST

The authors declare having no competing interests, financial or otherwise, and no professional relationships with companies or manufacturers who may benefit from the results of the present study.

## Data Availability

The data that support the findings of this study are available from the corresponding author upon reasonable request.
